# More intravascular volume, less edema: pressure therapy for the management of capillary leakage and fluid accumulation

**DOI:** 10.1186/s13613-025-01547-8

**Published:** 2025-09-02

**Authors:** Auguste Dargent, Vanessa Louzier, Jean-Pierre Quenot

**Affiliations:** 1 Department of Anesthesia and Intensive Care, Lyon Sud Teaching Hospital, Lyon, France; 2 Université de Lyon, UR APCSe Agressions Pulmonaires et Circulatoires dans le Sepsis, VetAgro Sup, 1 avenue Bourgelat, Marcy l’Etoile, F-69280 France; 3 Medical Intensive Care Department, CHU Dijon, Dijon, 21000 France

Dear Editor,

We have read with great interest the study by Posch et al. [1] in Annals of Intensive Care and congratulate the authors for their work. The study demonstrated that manual decongestive therapy (MDT) resulted in an increase in the global end-diastolic index (GEDI), occurring as fast as 15 min and sustained over 24 h. This increase in cardiac preload was associated with a decrease in limb edema without increasing fluid intake, suggesting that fluid can be redistributed into the vascular system using this technique. As noted by the authors, our group realized the first trial of pressure therapy using compression bandages to prevent fluid overload in patients with septic shock [2]. As the authors, we believe that pressure therapy has the potential to reduce and/or prevent fluid overload and improve the outcome of critically ill patients. However, we have some comments on the work by Posch et al. and the future perspectives of pressure therapy to address fluid overload.

First, we would like to comment on the nature of the intervention in this study, which consisted of two different phases. Manual drainage itself was performed first, followed by the application of short-stretch bandages. This is important because pressure therapy may result in two distinct types of effects. Indeed, as stated by the authors, pressure will act on Starling forces (the applied pressure limits transcapillary filtration and enhances lymph flow) but may also increase the proportion of stressed venous blood volume by compression of large venous reservoirs in the limbs. The latter mechanism is based on the principle of anti-shock trousers and has been used as a preload-dependency test [3]. However, very high pressure is required to deplete the deep veins, and anti-shock trousers are inflated to 80 mmHg [3]. In contrast, the short-stretch bandages used in the present study were the same as those in our study and were designed to apply low interface pressure (a median of 17 mmHg was measured in our pilot trial). Thus, in our opinion, it is very unlikely that venous compression occurred with these bandages. The interface pressure, which was not reported here, should be recorded in future pressure therapy trials.

The observed effect on GEDI may be due, for the most part, to an increase in plasma volume via lymphatic return. This hypothesis may have been confirmed by a decrease in the hematocrit (e.g., from blood gas samples, possibly at the author’s disposal? ). In our opinion, the early effect of MDT on GEDI is probably related to the manual, “active” part of the treatment (*intermittent and high* pressure), whereas the sustained effect observed after 24 h may demonstrate the role of *continuous and low* pressure in preventing further accumulation in the interstitium. It would be very interesting to distinguish the effects of these complementary pressure-based approaches: the authors could have used MDT and bandages in a randomized crossover, for example, although more patients would have been required.

The other issue we would like to discuss is the timing of the intervention. The presence of edema was the main inclusion criterion, and the median delay was–5–6 days since admission. Thus, it is safe to assume that most patients suffered some degree of fluid accumulation syndrome, although the cumulative fluid balance since admission was lacking from the baseline characteristics. In the Resuscitation, Optimization, Stabilization, and Evacuation (ROSE) framework introduced to apprehend the management of fluid overload, these patients would probably be classified in the stabilization or evacuation phase. Whether an increase in cardiac preload is a relevant outcome in this context is debatable. The study was not powered to detect a decrease in fluid balance, but we believe it may be a better target outcome for future trials. We also believe that pressure intervention should be performed as early as possible in high-risk patients to prevent fluid accumulation syndrome and to take advantage of Laplace’s law (compression is less efficient when the limb circumference increases). This “preventive” strategy is especially relevant, as the interstitium can decrease its hydrostatic pressure to enhance fluid filtration [4]. Although the role of interstitial pressure in fluid accumulation is difficult to demonstrate, it is probably an early mechanism.

In conclusion, the effect of pressure therapy as suggested in this study could be summarized as “more circulating volume with less oedema” which happens to be exactly what we desperately hope for in patients with fluid accumulation and concurrent hypovolemia [5]. We encourage the authors of this brilliant study and others to perform further studies on pressure-based therapies aimed at decreasing fluid overload in critically ill patients.

## Data Availability

Not applicable.
